# Retraction: Nuclear factor kappa-B signaling is integral to ocular neovascularization in ischemia-independent microenvironment

**DOI:** 10.1371/journal.pone.0227327

**Published:** 2019-12-30

**Authors:** The PLOS ONE Editors

After this article [1] was published, concerns were raised about several of the reported results.

Specifically:

- In the upper left panel of Fig 2, there are several regions that appear similar to one another.
- In Fig 2 the image in row 2, column 1 is reported as a high magnification view of the Control C57BL/6 Mice image in row 1, column 1, but it appears instead to be a higher magnification view of the YC-1-Treated rho/VEGF Mice image (row 3, column 3).
- In Fig 2, there are several elements that appear similar within and across the high magnification images of the Non-Treated rho/VEGF Mice, DMSO-Treated Rho/VEGF Mice, and YC-1-Treated rho/VEGF Mice.
- In Fig 6 (SDF-1 panels), Fig 8 (column 1, rows 1 and 4; column 2, rows 2, 3, 4), and Fig 9 (column 1, rows 2, 3, 4; column 2, row 4), several areas within layers appear similar.
- In Fig 7, similarities were noted between the following image pairs. In each case there are differences between the images in question as well as unexpected repetitions of image details:
  ○ column 1, row 2 and column 2, row 3
  ○ column 1, row 4 and column 2 row 1
- In Fig 7, similarities were noted within the images shown in column 1, row 4 and column 2, row 3.

The authors were unable to resolve these issues or provide the original data underlying the results in response to journal queries. Without the original data we cannot resolve the above concerns which call into question the integrity of the images and the validity of the reported results.

In light of these concerns, the *PLOS ONE* Editors retract this article.

Per FAAM, the underlying data for this study were held by MD, for whom current contact information is not available. The journal has not received confirmation that MD received communications about this case.

FAAM replied to our notification but did not specify agreement or disagreement with the retraction. MD either could not be reached or did not reply.
